# Randomized Clinical Trial Comparing Bare-Metal Stents Plus Colchicine Versus Drug-Eluting Stents for Preventing Adverse Cardiac Outcomes: Three-Year Follow-Up Results of the ORal Colchicine in Argentina (ORCA) Trial

**DOI:** 10.3390/jcm14092871

**Published:** 2025-04-22

**Authors:** Alfredo Matias Rodriguez-Granillo, Juan Mieres, Carlos Fernandez-Pereira, Camila Correa Sadouet, Jose Milei, Sandra Patricia Swieszkowski, Pablo Stutzbach, Omar Santaera, Pedro Wainer, Juan Rokos, Camila Gallardo, Roberto Cristodulo-Cortez, Ricardo Perez de la Hoz, Adnan Kastrati, Alfredo E. Rodriguez, on behalf of ORCA Investigators

**Affiliations:** 1 Otamendi Hospital, Buenos Aires 1001, Argentina; mrodriguezgranillo@gmail.com (A.M.R.-G.); jmieres@gmail.com (J.M.); cfernandezpereira@centroceci.com.ar (C.F.-P.); camilacorreasadouet@gmail.com (C.C.S.); wainerp@otamendi.com.ar (P.W.); juanmrokos@gmail.com (J.R.); gallardocami@gmail.com (C.G.); perezdelahozr@otamendi.com.ar (R.P.d.l.H.); 2 Centro de Estudios en Cardiología Intervencionista (CECI), Buenos Aires 1024, Argentina; 3 Sanatorio Las Lomas, San Isidro 3031, Argentina; p.stuzbach@gmail.com (P.S.);; 4 Hospital de Clinicas, Universidad de Buenos Aires, Buenos Aires 1113, Argentina; josemilei@gmail.com (J.M.); swwieszkowski@sanatorio-otamendi.com.ar (S.P.S.); 5 Hospital Obrero, No 2–CNS, Santa Cruz, Bolivia; achesini@centroceci.com.ar; 6 Deutsches Herzzentrum, 80636 Munich, Germany; kastrati@dhm.mhn.de

**Keywords:** colchicine, stents, drug-eluting stents, acute myocardial infarction, angiographic score risk, acute coronary syndromes

## Abstract

**Background**: In patients with coronary artery disease, bare-metal stents (BMS) are considered a safer but less effective treatment than drug-eluting stents (DES). Oral colchicine therapy may compensate for this limitation of BMS. This randomized trial compared the cost-effectiveness of two different revascularization strategies during percutaneous coronary intervention (PCI). **Methods**: Between March 2020 and April 2022, 410 patients were randomly treated with PCI with BMS plus colchicine (BMS-CO: 205 patients) or DES (205 patients) The patients in the BMS-CO group received 0.5 mg oral doses of colchicine for 3 months. The primary endpoint was major adverse cardiac and cerebrovascular events (MACEs), defined as the composite of death, myocardial infarction, stroke, or target vessel revascularization (TVR), and the costs of each treatment strategy. The secondary endpoints included the individual components of MACEs. **Results**: No significant differences were observed in baseline characteristics, and 76% of the patients presented with acute coronary syndromes. The median follow-up period was 36.8 months. Five percent of the patients in the BMS-CO group discontinued study medication. The cumulative incidence of MACEs was not significantly different, with 12.7% in the BMS-CO group and 15.6% in the DES2G group (*p* = 0.39) as well individual components of the clinical endpoint. The cumulative costs were lower in the BMS-CO group than in the DES2G group (USD 4826.4 ± 2512 vs. USD 5708 ± 3637, *p* < 0.001). **Conclusions**: In the 3 years, the DES strategy failed to be cost-saving compared to BMS-CO. However, due to the small sample size, the equivalence in clinical outcomes with both strategies can occur by chance (NCT04382443).

## 1. Introduction

Since their introduction into clinical practice, drug-eluting stents (DES) have become the standard of care for percutaneous coronary intervention (PCI) due to their ability to reduce in-stent restenosis (ISR) and improve clinical outcomes compared with bare-metal stents (BMS) [1,2,3,4,5]. The new-generation DES (DES2G) have further improved outcomes and reduced the risk of stent thrombosis [6]. The advantages of DES2G over BMS in several clinical outcomes were confirmed by a meta-analysis based on individual patient data and were limited to the first year after the procedure [5] and lesion localization [7] in the left main coronary artery (LMCA) or the left anterior descending artery (LAD).

Inflammation plays an important role in atherosclerosis and the pathogenesis of acute coronary syndromes (ACSs) [8,9]. In previous large randomized controlled trials (RCTs), the use of colchicine was associated with a significant reduction in major adverse cardiac events (MACEs) in patients with chronic coronary syndromes (CCSs) or ACSs [10,11,12]. The potential role of adjunctive colchicine in reducing ISR was evaluated in a small randomized trial in diabetic patients, in which colchicine showed a significant reduction in ISR compared to BMS alone [13]. However, studies with DES2G are lacking.

In several small RCTs performed in the past, oral anti-inflammatory or immunosuppressive drugs after BMS implantation showed similar results in terms of target vessel revascularization (TVR) and myocardial infarction (MI) compared to DES. However, no comparison has been made with the new-generation DES [13,14,15,16,17,18,19,20,21].

We report here the 3-year results of the ORCA (ORal Colchicine in Argentina) randomized trial, which compared a strategy of DES2G versus BMS with three months of colchicine [22,23]. The preliminary and two-year follow-up data were reported in abstract form in 2023 at the American Heart Association meeting.

## 2. Material and Methods

### 2.1. Trial Design

The ORCA trial was an investigator-initiated, multicenter, prospective, open-label, randomized controlled clinical trial (ClinicalTrials.gov identifier: NCT04382443) [22] performed in 2 centers in Argentina and one center in Bolivia; see Supplementary Materials.

The study received no private or public funding. The study was conducted in accordance with good clinical practice and the tenets of the Declaration of Helsinki and was approved by the research and ethics committee and the medical director of each center. The protocol and informed consent were approved by an independent ethics and review board (IERB) on 10–11 February 2020. National Health Authorities (ANMAT) also approved the protocol (12 February 2020; approval code: MN.60090). ANMAT, which is part of the Argentinian Ministry of Health, controls medications and clinical trials in Argentina. Modified versions of the protocol and informed consent allowing the inclusion of patients older than 80 were approved in September 2020. The centers involved in the study approved the protocol and patient inclusion. All patients signed an informed consent before enrollment. An independent data safety and clinical events committee (DSC and CEC, respectively) monitored and blindly adjudicated events in each patient. The regulatory steps and the study organization are described in detail in the Supplementary Materials.

### 2.2. Participants, Randomization, and Follow-Up

The patient screening started immediately after ANMAT and the IERB approved the study protocol and informed consent in the last week of February, which is the rule for all randomized trials in Argentina. The first patient was randomized and treated in March 2020, concomitant with the trial registration (NCT04382443).

All patients referred to the Cath Lab service for coronary angiography and the indication for myocardial revascularization were eligible for this study. The study’s major inclusion and exclusion criteria are in the Supplementary Materials. The randomization sequence was carried out in blocks of 10 and placed in sealed envelopes drawn before the start of the study. The patients were allocated to an established treatment as DES2G (DES2G group) versus BMS, followed by three months of colchicine, 0.5 mg twice daily, (BMS-CO group) as the comparator. The DES2G group did not receive adjunctive colchicine.

The stent designs for each group are described in the Supplementary Materials.

**Figure 1:**
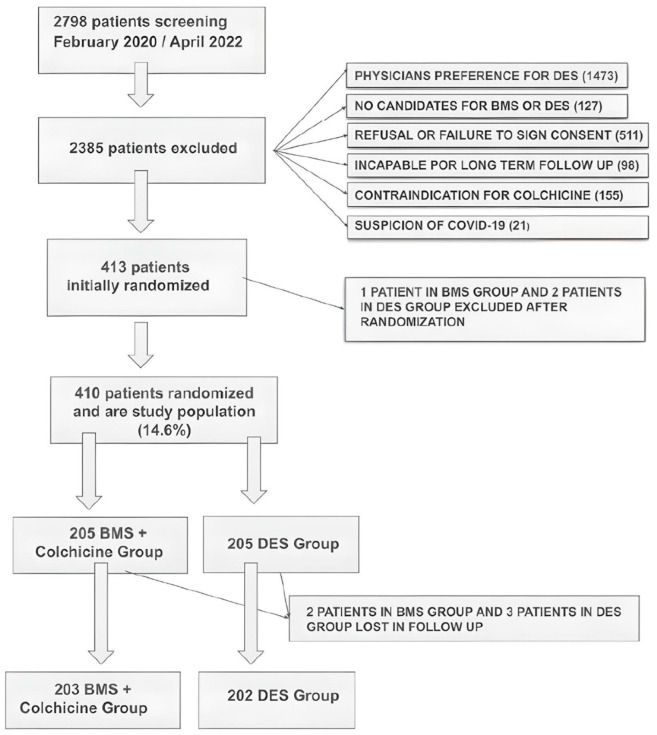
Study design and patient population.

Multiple vessel disease was defined as the presence of lesions with ≥70% diameter stenosis as determined by visual estimation in 2 or 3 major epicardial vessels defined as multiple vessel disease. The flow diagram of the study is shown in Figure 1.

### 2.3. Procedure and Follow-Up

All patients in the DES2G group received dual antiplatelet therapy (DAPT) with a P2Y12 inhibitor (clopidogrel, prasugrel, or ticagrelor) and 100 mg of aspirin daily. Although most patients continued DAPT for 12 months after the index procedure, regardless of their clinical status at enrollment, a shorter duration of DAPT was also allowed, based on clinical practice in Argentina and Bolivia [24,25,26].

For patients with ACS, prasugrel and ticagrelor were the preferred drugs according to current guidelines [24,25,26]. In the BMS-CO arm, patients with CCS received 30 days of DAPT followed by 100 mg of aspirin for one year. Patients with ACS received DAPT for six months and 100 mg of aspirin for one year. Daily doses were 75 mg for clopidogrel and 10 mg for prasugrel; ticagrelor was given 90 mg twice a day. All patients received statins.

Lesion complexity was angiographically categorized according to SYNTAX (SS) and ERACI Scores (ES). Both scores are described in the Supplementary Materials [27,28,29].

After hospital discharge, clinical interviews were conducted at 1 month and at 3, 6, 9, and 12 months. Subsequently, patients were contacted in person or by telephone at 2, 3, 4, and 5 years. If a cardiovascular event was reported, trained personnel at the study’s coordinating center reviewed the complete source documentation.

The study was monitored by the CECI (Centro de Estudios en Cardiologia Intervencionista).

### 2.4. Endpoint Definitions

The primary endpoints were defined as a composite: (1) Major adverse cardiac and cerebrovascular events (MACEs) were defined as the composite of all-cause death, procedural and non-procedural myocardial infarction (MI), cerebrovascular accident (CVA), and ischemia-driven target-vessel revascularization (TVR). (2) Cumulative costs, assuming no significant difference in MACEs, were also part of a primary endpoint as a cost-effectiveness hypothesis.

The composite primary endpoint was described in the clinicaltrials.gov site of the ORCA trial and in the study protocol published in 2020 [22,23].

Other endpoints were target lesion failure (TLF), defined as cardiac death, MI, and ischemia-driven revascularization of the target lesion (TLR). Non-TVR was also analyzed.

MI was defined as periprocedural during PCI or related to follow-up revascularization. Spontaneous MI was defined as any MI occurring beyond seven days from initial PCI to the last follow-up.

MI was defined as an increase in cardiac biomarkers (CK-MB or troponin) ≥5 times above the upper limit of normal, with either evidence of prolonged chest pain or ischemic ST-segment changes or new pathological Q waves or noninvasive evidence of new regional wall motion abnormality. Detailed MI definitions are shown in the previous publication on study design and Supplementary Materials. Detailed MI definitions are shown in the previous publication of the study design [23] and in the Supplementary Materials.

We planned to assess changes in biological markers of inflammation; this was also performed in patients with ACS. Blood samples were collected on the day of angioplasty and at 30 days thereafter for measurement of C-reactive protein (CRP) in both study arms.

### 2.5. Statistical Analysis

The study’s sample size was calculated based on a test for a trend analysis. A two-sided test for differences with an alpha error of 0.05 was used to determine the power to detect a significant difference in overall cost between treatment groups at 1 year, assuming that the MACEs were similar in both groups.

Based on previous colchicine data and our experience with oral immunosuppressive therapy (OIT) plus BMS implantation, the incidence of TVR and MACEs would be similar in the DES2G and BMS-CO groups [13,14,15,16,17,18,19,20,21]. Therefore, the sample size was calculated based on differences in cost between the 2 PCI strategies.

In all the randomized trials, including one with colchicine, using immunosuppressive or anti-inflammatory drugs, such as rapamycin or prednisone after BMS implantation, significantly reduced TVR and TLR compared to either BMS alone or first-generation DES. This was summarized and highlighted in an individual patient-level meta-analysis published in 2014 of all the randomized clinical trials published at that time, which included 1246 patients [21]. BMS + OIT reduced the risk of revascularization 0.49 [0.24–0.98], *p* = 0.04, versus BMS and was not significantly different from DES 1.49 [0.50–4.07], *p* = 0.50.

Considering the clinical equivalence between both strategies, a non-inferiority test was based on the previous cost-effective analysis of oral rapamycin plus a BMS versus DES trial [17,18,19,20]. A one-tailed noninferiority test was based on a predetermined noninferiority threshold level of 15% with an overall alpha < 0.05 and was validated by the bootstrap method [17,18,19,30].

The hypothesis was that the average DES costs minus the average costs of colchicine plus BMS were greater than the prespecified noninferiority threshold [18].

The cost of colchicine was added to the 3-month follow-up cost.

Costs associated with new adverse events during follow-up were added if they were related to the initial procedure or the progression of coronary artery disease. Colchicine costs are negligible worldwide; the same is true in Argentina and Bolivia. The Supplementary Materials describe more cost analyses.

We determined that the enrollment of at least 400 patients (200 in each group) was required.

Continuous variables were expressed as mean ± SD and categorical variables as percentages (%). ANOVA with Bonferroni correction was used for continuous variables, and chi-square analysis or Fisher’s exact test for categorical variables. A Kaplan–Meier curve was used for survival analysis, and the incidence corresponded to Kaplan–Meier estimates. Hazard ratios (HR) and their 95% confidence intervals (CIs) were calculated with the use of a Cox proportional hazard model. Statistical analysis was performed with SPSS v. 25.0, and a *p*-value < 0.05 was considered to demonstrate statistical significance.

All events were analyzed and adjudicated by the intention-to-treat principle.

## 3. Results

From February 2020 to April 2022, 2798 patients were screened and randomized in the three participating centers of the ORCA trial: Otamendi Hospital, Sanatorio Las Lomas, in Argentina, and Hospital Obrero, in Bolivia. Of them, 2385 patients were excluded; the reasons for the exclusions are described in the Supplementary Materials and Figure 1. Four hundred and thirteen patients were initially randomized and entered into the study. The DSC excluded three patients who developed severe COVID-19 during initial hospitalization: one in the BMS-CO group and two in the DES2G group. Therefore, 410 patients entered the trial and represented the study population (205 in each group). All randomized patients received the assigned treatment. Long-term follow-up was obtained in 99% and 98.5% of patients in BMS+CO and DES2G, respectively (Figure 1).

Table 1 shows baseline demographic and clinical characteristics. No significant differences were observed between the two groups. Many patients presented with ACS (76%) and ST-segment elevation MI (STEMI, 22%).

Angiographic and procedural characteristics were also evenly distributed between the two groups (Table 2). Multivessel disease was present in 45% of the patients. A target lesion was localized in LMCA in 5% of the cases and in the LAD in 58% of the patients. The mean SYNTAX score (SS) and ERACI score (ES) were 21.4% and 15.2%, respectively. The number of stents per patient was, on average, 1.61 and 1.57 in the DES2G and BMS-CO groups, respectively. The stent type is described in the SA. All DES belonged to the latest generation and were approved by local regulatory agencies for clinical use.

In the BMS-CO group, 5% developed adverse side effects related to colchicine therapy (diarrhea in all), most of them mild, and had to discontinue the treatment—only one needed hospitalization for three days.

### 3.1. Three-Year Follow-Up Results

The median follow-up was 36.8 months (range 23 to 47 months).

At 1 year, 71% of the BMS-CO patients and 89% of the DES2G patients took P2Y12 inhibitors; at 3 years, these figures were 21% and 43%, respectively (*p* < 0.0001).

**Figure 2:**
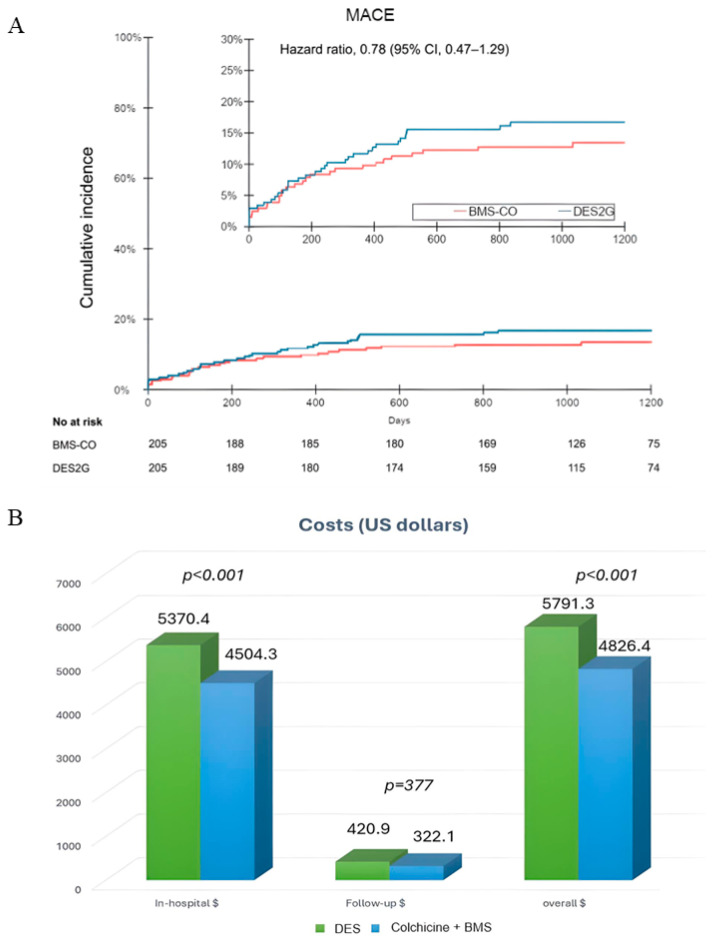
(**A**) Freedom From MACEs in both groups. (**B**) Hospital, follow-up, and overall cost. BMS: bare-metal stents, CO: colchicine, DES2G: drug-eluting stents 2nd generation.

The clinical outcomes are shown in Table 3. At three years, the incidence of the primary composite endpoint of the MACEs was15.6% in the DES2G and 12.7% in the BMS-CO arm (HR, 0.78 [95% CI, 0.47–1.30], *p* = 0.39); Figure 2A and central illustration. At three years, the mean cumulative costs were significantly lower in the BMS-CO (USD 4826.4) than in the DES2G group (USD 5791.3), *p* = 0.0001; Figure 2B. The cost differences between the treatment strategies reflect the initial cost differences between BMS and DES2G, the negligible costs of the colchicine, the reduced costs of a shorter P2Y12 therapy in the BMS-CO group, and the lack of differences in the need for revascularization between the two groups of shorter duration; in the Supplementary Materials Table S1, the cost of the initial procedure is described, including the follow-up and cumulative cost. The overall cost difference between both treatment arms was USD −964.9, *p* < 0.001.

**Figure 3:**
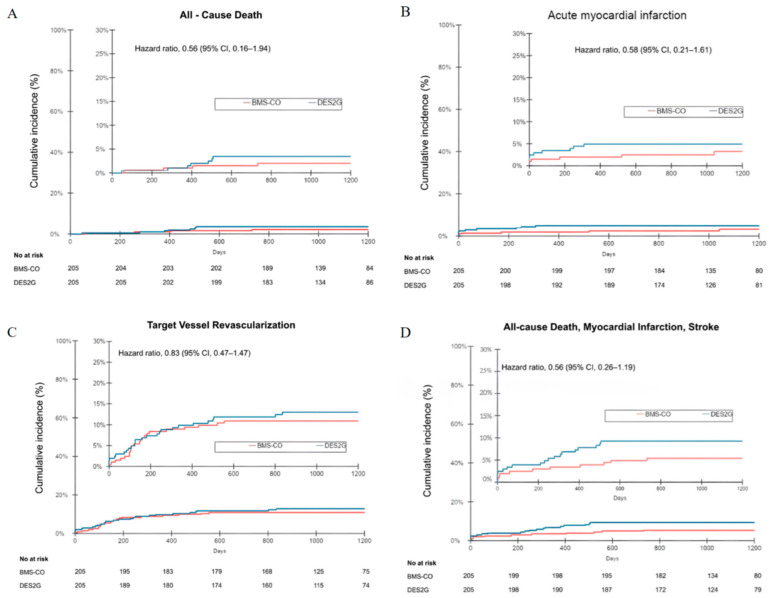
(**A**) All causes of death; (**B**) incidence of myocardial infarction; (**C**) incidence of target vessel revascularization; (**D**) incidence of all causes of death, myocardial infarction, stroke. BMS: bare-metal stents, CO: colchicine, DES2G: drug-eluting stents 2nd generation.

No significant differences were observed regarding all other outcomes between the two groups (Table 3). The incidence of TLF was 12.6% in the DES2G and 12.3% in the BMS-CO arm (HR 0.83, 0.47–1.47, *p* = 0.61). More specifically, all-cause death (Figure 3A), MI (Figure 3B), ischemic TVR (Figure 3C), and the composite of death, MI, and CVA (Figure 3D) showed no significant differences between the two groups.

Cox logistic regression found that age and residual ERACI score are related to lower MACEs. Supplementary Materials Table S2 describes a detailed analysis.

### 3.2. Changes in Inflammatory Markers

**Figure 4:**
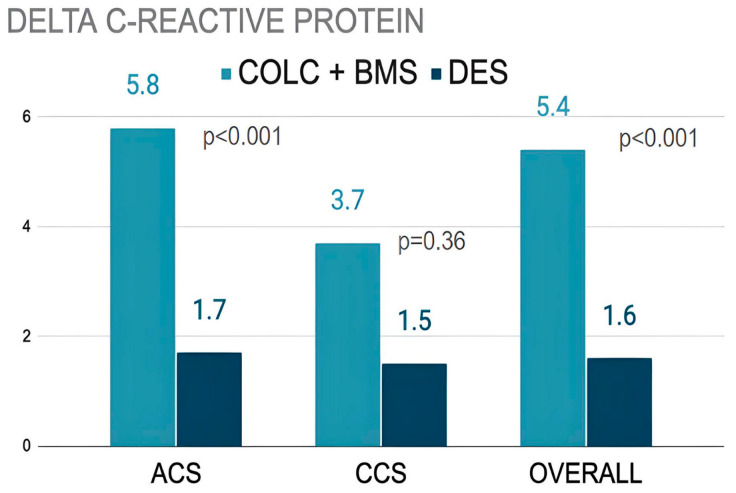
Difference in CRP levels at baseline and 30 days. BMS: bare-metal stents, CO: colchicine, DES2G: drug-eluting stents 2nd generation. ACS: acute coronary syndrome, CCS: chronic coronary syndromes.

We measured baseline and 30-day CRP levels as part of the prespecified analysis in 189 patients. All the patients in the BMS-CO group had a full 30-day therapy with colchicine. There was a significantly greater reduction in CRP (delta change) from baseline to 30 days in the BMS-CO group (5.4 mg/L) than in the DES2G group (1.6 mg/L), *p* < 0.001, and differences were observed in all the clinical scenarios, but they were significantly greater in the ACS driving for the sample size (Figure 4). The mean value of the CRP levels at 30 days in BMS + CO was 1.7 mg/L ± 3.7.

## 4. Discussion

In this randomized comparison of two revascularization strategies during PCI, the use of BMS implantation followed by an oral daily dose of 1 mg of colchicine for three months achieved similar clinical outcomes at three years of follow-up compared with a strategy of PCI with an established approach of implantation of new-generation DES alone with the advantage of reduced costs.

These findings highlighted the role of colchicine as an anti-inflammatory drug, suggesting that it interferes with the process of ISR and compensates for the disadvantage of BMS in this regard.

Colchicine was used several years ago as an anti-inflammatory and immunosuppressive drug to treat acute gout attacks [10], and randomized trials have recently demonstrated that it improves clinical outcomes in patients with acute and chronic CAD [10,11,12,31,32,33,34].

In one randomized trial, BMS implantation plus six months of colchicine was associated with a significant reduction in restenosis compared with BMS plus a placebo in diabetics [13]. Several small RCTs in the past have systematically shown the benefit of a short oral immunosuppressive therapy after BMS implantation compared to BMS alone or first-generation DES designs [14,15,16,17,18].

In two more recent randomized trials, colchicine was given at single bolus doses before PCI. In the study by Cole and colleagues that included 75 patients, colchicine reduced periprocedural myocardial injury [31,32]. In the study by Shah and colleagues that included 400 patients, colchicine was not able to improve clinical outcomes, although it reduced the inflammatory markers at 24 h after PCI [33]. However, the (cost-)effectiveness of colchicine has not been assessed against second-generation DES.

In the recently reported large randomized study, Clear Synergy [34], the addition of colchicine did not improve clinical outcomes. We can only speculate about differences, but we have two main hypotheses for the positive results in ORCA against those negative results in Clear Synergy:

First, the stent design was BMS in ORCA in 100% of cases vs. 0.3% in Clear Synergy.

Intimal hyperplasia during the first year was the main limitation of the BMS design, and colchicine was demonstrated to reduce it in a randomized clinical trial significantly [13]. In contrast, endothelial dysfunction and early neo-atherosclerosis observed after DES implantation, were regarded as the main limitation of the current DES2G. To our knowledge, the lack of vasomotion observed after DES implantation could not be reversed with the use of anti-inflammatory drugs such as colchicine [35,36,37].

Secondly, we gave 0.5 mg twice a day, which is double the dose recommended in Clear Synergy. Even though CRP was significantly reduced, it was still at a high therapeutic level of 3 mg/L, which is far from the goal of below 2 mg/L reported by the sub-analysis of the large randomized trial of Canakinumab to analyze CRP reduction in improving clinical outcomes [38]. In contrast, in ORCA, the CRP level at 30 days in colchicine was 1.4 mg/L.

In Clear Synergy, if we look at the data in the forest plot in Figure 2, where patients were taking colchicine twice a day, there is a strong trend in favor of colchicine 0.78, 0.61–1.00 [34].

It is well known that the latest generation of DES has significantly improved safety and efficacy compared to the first DES or BMS and has become the default strategy during PCI.

However, the most extensive RCTs, NORSTENT (Norwegian Coronary Stent Trial), did not show a survival benefit with DES2G compared to BMS [39]. Also, a recent meta-analysis from RCTs showed only a safety benefit of DES2 over BMS in patients with LMCA or LAD lesions [7]. Conversely, it did not show a benefit in non-LMCA/LAD lesions, where all-cause death at five years was numerically higher with DES2G (7). In addition, some low- and mid-income regions, including Latin America, still have economic restrictions that prevent the large use of DES, as was reported in a recent regional registry [40].

Of note, despite a significant reduction in repeat TVR and TVR-MI with DES, it has not improved the benefit of PCI against CABG or optimal medical treatment, as seen in all RCTs performed in the last 20 years [41,42,43,44,45,46,47,48,49,50]. Moreover, in the most recent meta-analysis of dedicated RCTs, the incidence of death and spontaneous MI was significantly higher with DES compared to CABG [50]. Lesion complexity and differences in the completeness of revascularization cannot explain these findings. In the NOBLE trial (Nordic–Baltic–British left main revascularization study), DES2G and CABG achieved similar completeness of revascularization, but spontaneous MI was significantly higher with PCI [49]. Similarly, CABG was superior to FFR-guided PCI in the FAME 3 trial (Fractional Flow Reserve versus Angiography for Multivessel Evaluation), which included a low-risk population [41,42].

Early neo-atherosclerosis and endothelial dysfunction could be linked with the above findings, and both remain as limitations of DES2G [35,36,37].

## 5. Limitations of the Study

The study had a few limitations.

First, the sample size is underpowered to detect differences in all clinical endpoints. The concern regarding this interpretation of the result would be primarily related to a lack of any statistical significance in terms of non-inferiority between BMS and DES. Since the cost of older-generation devices tends to be lower, the cost difference in the two strategies primarily stems from the difference in the cost of the procedure, which is to state that if longer-term follow-up or a larger sample size shows that the BMS and colchicine arm requires further repeat procedures, the initial cost benefit would be negated.

Had there been a statistically significant non-inferiority demonstrated, one could extrapolate the cost benefit to be favoring the BMS plus colchicine approach. However, most of the differences in RCT between BMS and DES are restricted during the first year after deployment [5,7]; furthermore, in our study, no differences were seen during late follow-up on TVR between both strategies, in agreement with previous experiences with OIT after BMS implantation [16,17,18,19].

The low number of patients with baseline and 30-day CRP samples reflected the study’s non-sponsored nature and local Medicare’s budget limitations.

Secondly, colchicine was given in the BMS arm only.

We recognize that it would also be reasonable to give colchicine in the DES2G arm; however, the significant limitations of DES2G are endothelial dysfunction and early neo-atherosclerosis [42,43,44], and to our knowledge, none of them would be prevented within three months of colchicine. We previously discussed the results of Clear Synergy above.

The study was not blinded, but all the clinical events underwent blinded adjudication.

Only 14.7% of the recruited patients were randomized (Supplementary Materials). Therefore, when analyzing ORCA results, we should consider their baseline clinical and angiographic characteristics, including many ACS and STEMI patients and low angiographic risk scores. These findings cannot be extrapolated to more complex lesion subsets or clinical scenarios.

## 6. Conclusions

At the 3-year follow-up, due to no differences in effectiveness between the two revascularization strategies, the DES strategy failed to be cost-saving and effective compared to BMS plus oral colchicine.

The findings of this study strengthen previous reports on the value of OITs, including colchicine, in preventing TVR and MACEs after BMS implantation.

However, the study’s sample size was only powered for cost-saving differences. Therefore, the similarities in all clinical outcomes between the BMS plus colchicine and the DES2G groups should be interpreted cautiously, and we cannot discard the fact that they might have occurred by chance. Furthermore, clinical and angiographic scenarios of this trial, including large numbers of patients with ACS and low angiographic score risk should be considered when analyzing the results.

## Figures and Tables

**Table 1:** Baseline clinical and angiographic characteristics.

Variable	Colchicine + BMS	DES	*p*-Value
N	205	205	
Age	64.6 ± 11.7	64.9 ± 11.4	0.82
Male gender, %	86.3	82	0.22
HBP, %	65.9	73.6	0.63
Dyslipidemia, %	53.2	52.4	0.83
Smoker, %	24.9	25.7	0.84
Diabetes, %	19.0	21.8	0.47
Previous MI, %	13.7	19.4	0.11
Acute Coronary Syndrome at baseline, %	78.0	74.6	0.25
STEMI, %	22.9	20.9	0.63

BMS = bare-metal stents; DES = drug-eluting stents; HPB = high blood pressure; MI = myocardial infarction; STEMI = ST elevation myocardial infarction.

**Table 2:** Angiographic characteristics.

Variable	Colchicine + BMS	DES	*p*-Value
N	205	205	
Treated vessels per patient	1.22 ± 0.4	1.25 ± 0.5	0.59
Stents per patient	1.57 ± 0.7	1.61 ± 0.8	0.56
ULMD, %	4.9 (10)	5.4 (11)	0.82
LAD, %	57.6 (118)	57.6 (118)	0.92
MVD, %	44 (90)	46.3 (95)	0.62
SYNTAX score	21.8 ± 11.4	21.1 ± 9.4	0.49
Residual SYNTAX score	9.3 ± 7.8	8.1 ± 7.5	0.24
ERACI score	15.4 ± 9.3	15.1 ± 8.6	0.82
Residual ERACI score	3.3 ± 5.8	2.5 ± 6.3	0.35
Clopidogrel, %	39.2 (80)	38.2 (78)	0.84
Radial access, %	46.8 (96)	35.6 (73)	0.21
Intravascular imaging	2.4 (5)	2.9 (6)	0.76

BMS = bare-metal stents; DES = drug-eluting stents; ULMD = unprotected left main disease; LAD = left anterior descending artery; MVD = multiple vessel disease.

**Table 3:** Clinical outcomes at follow-up.

Variable	Colchicine + BMS	DES	Hazard Ratio (95%)
N	205	205	
Primary endpoint, %	12.7 (26)	15.6 (32)	0.78 (0.47–1.29)
Overall death, %	2.0 (4)	3.4 (7)	0.56 (0.16–1.94)
Cardiac death, %	1 (2)	2 (4)	0.49 (0.09–2.71)
Myocardial Infarction, %	2.9 (6)	4.9 (10)	0.58 (0.21–1.61)
Periprocedural MI, %	1.0 (2)	2.4 (5)	0.25 (0.028–2.23)
Spontaneous MI, %	2.0 (4)	2.4 (5)	0.81 (0.24–2.67)
CVA, %	0.5 (1)	1.0 (2)	0.50 (0.04–5.56)
TVR, %	9.5 (24/251)	9.4 (24/256)	0.83 (0.47–1.47)
All cause of death, MI, Stroke, %	5.4 (11)	9.3 (19)	0.56 (0.26–1.19)
TLF, %	12.3 (36)	12.6 (38)	0.83 (0.47–1.47)
P2Y12 Inhibitors	21 (43)	43 (88)	1.73 (1.25–2.4)

BMS = bare-metal stents; DES = drug-eluting stents; MI = myocardial infarction, CVA = cerebrovascular accident; TVR = target vessel revascularization; TLF = target lesion failure.

## Data Availability

Please contact the first or corresponding authors by mail or go to clinical trials.gov reference [22].

## Supplementary Materials

The following supporting information can be downloaded at https://www.mdpi.com/article/10.3390/jcm14092871/s1.

## Abbreviations and Acronyms

ISR	In-stent restenosis
LMCA	Left main coronary artery
LAD	Left anterior descending artery
DES	Drug-eluting stents
DES2G	Latest DES designs
BMS	Bare-metal stents
CO	Colchicine
DSC	Data safety committee
CEC	Clinical events committee
IERB	Independent Ethical Review Board
ACS	Acute coronary Syndromes
CCS	Chronic coronary syndromes
AMI	Acute myocardial infarction
SS	SYNTAX Score
ES	ERACI Score
CAD	Coronary artery disease
MACEs	Major adverse cardiovascular events
TLF	Target lesion failure
TVR	Target vessel revascularization
CVA	Cerebrovascular accident
ORCA	ORal Colchicine in Argentina
MI	Myocardial infarction
OIT	Oral immunosuppressive therapy
