# Supplementary material for: Randomized Clinical Trial Comparing Bare-Metal Stents Plus Colchicine Versus Drug-Eluting Stents for Preventing Adverse Cardiac Outcomes: Three-Year Follow-Up Results of the ORal Colchicine in Argentina (ORCA) Trial
Source: J Clin Med. 2025 Apr 22;14(9):2871. doi: 10.3390/jcm14092871 (PMC12072393; doi:10.3390/jcm14092871)
Supplement: Supplementary file 1 [file jcm-14-02871-s001.zip › jcm-3586595-supplementary.pdf]

Supplementary Appendix

Table 1 SA. In-hospital, Follow-up and Cumulative costs per patient in both groups

**Variables (US$)**	**BMS+Co**	**DES2G**	**p value**
Initial procedure	355+/-75	1694+/-965	<0.001
In-hospital costs (excluding procedure)	3485+/-2271	3478+/-2190	0.97
In-hospital costs	4504.3 +/-2278	5370+/-2356	<0.001
Follow-up	322.1 +/- 1143.5	420.9 +/-1293	0.36
Overall costs	4826.4 +/- 2452.2	5791.3 +/- 2700	<0.001

Table 2 SA. Identified independent variables of MACE (Cox Regression Analysis)

Name	p	HR	Lower 95% CI	Upper 95% CI
Age	,034	1,03	1	1,07
Residual Eraci score	,04	1,16	1,01	1,33
Group COLC	,635	1,16	0,64	2,1
Syntax score	,247	1,07	0,95	1,21
Eraci score	,298	0,93	0,82	1,06
Gender Male	,81	0,9	0,39	2,08
HBP No	,354	0,71	0,35	1,46
High cholesterol Yes	,882	0,96	0,53	1,74

Diabetes Yes	,484	0,77	0,36	1,62
Previous MI Yes	,605	1,21	0,59	2,51
STEMI No	,062	2,51	0,95	6,63
MVD Yes	,307	1,38	0,74	2,58

Study Organization

Study Chairs and Principal Investigator (PI) Alfredo E RodríguezMD, PhD; Co PI Carlos Fernandez Pereira MD,PhD and Professor Adnan Kastrati MD (Munchen, Germany).

Independent Ethical Committee: Comite de Etica en Investigacion Biomedica, Alberto C Taquini Universidad de Buenos Aires (Argentina).

Data safety monitoring committee: Jose Milei MD PhD, Sandra Swieszkowski MD, Hector Vetulli MD and Pedro Wainer MD

Clinical Events Committee: David Antoniucci, MD (CEC Chairperson, deceased) Careggi Hospital, Florence Italy); Valeria Curotto MD, Camila Correa-Sadouet MD, Eduardo Gabe MD (deceased).Jose Milei MD PhD, Sandra Swieszkowski MD

Angiocore Laboratory (Centro CECI): Juan Mieres MD, and Yasmin Navarro.

Blood Laboratory Data Analysis: Sanatorio Otamendi, Sanatorio Las Lomas

Biostatistics: Matias Rodriguez-Granillo MD,(Centro CECI)

Participating Centers: Carlos Fernandez-Pereira MD , Camila Correa-Sadouet MD,Diego Azcarrunz MD, Valeria Curotto MD, Dario Vita MD,Lucia Fontana MD, Camila Gallardo MD,Juan Rokos MD (Sanatorio Otamendi CABA,Argentina) , Juan MieresMD , Omar Santaera MD, Luciano Destefano MD, Pablo Stuzbach MD (Sanatorio Las Lomas, San Isidro Buenos Aires), Roberto Cristodulo-Cortez MD (Hospital Obrero, Santa Cruz de la Sierra, Bolivia).

Coordinating Center: Centro de Estudios en Cardiologia Intervencionista (CECI), a nonprofit organization of Centro CESI SA.

Alfredo M Rodriguez-Granillo MD, Monica Buus Lic, Anabel Chesini Lic, Carlos Biagioni , Chemist.

Study Regulatory steps.

1- Independent Ethical Review Board February 10, 2020.
2- Inform Consent February 11, 2020
3- Argentina National Health Regulatory Agency, February 12, 2020
4- Amendment of the Protocol, no age limit, September 20, 2020
5- The study organization made interim analysis at 12 and 18 months of follow-up.

Summary of primary clinical and angiographic eligibility criteria. (19)

Clinical and angiographic inclusion criteria

1-The subject must be ≥18 years old. .No age limit (after protocol amendment September 2020)

2. Patient (or legal guardian) indicates an understanding of the trial requirements and the treatment procedures and provides written informed

consent before procedures are performed

3. The patient has symptomatic coronary artery disease or silent ischemia with objective evidence of ischemia or acute coronary syndromes

4. Patient has one or more coronary artery stenosis of ≥70% in a coronary artery with a visually estimated reference vessel diameter ≥2.50 mm

5. A PCI procedure is indicated

6. Left ventricular ejection fraction >40% as measured within 60 days before enrollment

7. The patient is willing to comply with all follow-up evaluations required by the protocol

Clinical and angiographic exclusion criteria

1. The subject has a known allergy to contrast (that cannot be adequately premedicated) and/or the stent system or colchicine (e.g., cobalt–chromium

alloy, stainless steel, all P2Y12 inhibitors, aspirin)

2. Planned surgery within 30 days after index PCI

3. Short life expectancy(<36 months).

4. Drug abuse at the time of randomization.

5. The patient has a history of bleeding diathesis or coagulopathy or will refuse blood transfusions

6. The patient is participating in another investigational clinical trial and in the opinion of the investigator, may cause noncompliance with the protocol or confound data interpretation

7. The patient intends to participate in another investigational drug or device clinical trial within 12 months after the index procedure

8. The patient is a woman who is pregnant or nursing

9. DES restenosis from previous PCI interventions

10. Previous PCI with DES in the target vessel

11. The subject has an additional clinically significant lesion(s) in the target vessel for which an intervention may be required within 12 months after

the index procedure

12. The patient will be unavailable to close follow-up

Endpoint definitions.

Cardiovascular death includes sudden cardiac death, death due to acute MI, arrhythmia, heart failure, stroke, or bleeding

Non-cardiovascular death: any death with a known cause not of cardiac or vascular cause

Myocardial infarction (MI) Increase in cardiac biomarkers (CK-MB or troponin) >5× URL, with one of the following:

• Evidence of prolonged chest pain

• Ischemic ST-segment changes or new pathological Q waves

• Noninvasive evidence of new regional wall motion abnormality

Post-procedure MI: The occurrence of MI within seven days after PCI. with or without new pathologic Q-waves in at least two contiguous leads or new persistent left bundle branch block

Spontaneous MI: The occurrence of MI more than seven days after PCI

Each MI will also be classified as ST-segment elevation MI or non-ST-segment elevation MI.

Stent thrombosis is defined per the Academic Research Consortium criteria.

Major adverse cardiovascular events (MACE): Included death for any cause, MI, procedural and spontaneous cerebrovascular accident, and ischemic target vessel revascularization (TVR).

Target lesion failure included cardiac death, MI, and TVR.

Target lesion revascularization Repeat revascularization (including PCI and coronary artery bypass grafting) for target lesions in the presence of

symptoms or objective signs of ischemia.

All end-point definitions were previously described in the study protocol (19)

Cost Analysis

Cost differences in DES and BMS designs and long-term DAPT therapy requirements will drive cost variations (17). Costs (expressed in US dollars) included hospitalization for the initial procedure and follow-up new PCI procedural or related cardiac adverse events, medications (procedural and follow-up), and procedural resources (initial and follow-up). Professional fees during PCI procedures were estimated according to the national fees in Argentina and Bolivia.

Considering that both revascularization procedures share indirect costs, we only analyzed direct costs and cost differences between the two strategies using the microcosting method.

All direct costs from patients randomized in Argentina sites were actualized by the

Argentinean and Bolivia inflation rate and converted to US dollars in July 2024. Specific costing was done for each patient. The same stent list prices were used for allpatients. See in Table 1SA

Cox Regression Analysis of Predictor of Outcome

Logistic Cox regression analysis identifies age (HR CI 1.03 CI 1-1.07 p=0.034), high residual ES (HR 1.23 CI 1.00-1.34 p=0.04), and STEMI(HR 2.51 CI 0.95-6.6 p=0.06) as independent predictors of MACE at follow-up. All other baseline demographic, clinical, angiographic, or procedural characteristics didn't predict MACE at the longest available follow-up. The variables analyzed were sex, age, hypertension, diabetes, high cholesterol, renal failure, smoking, angina status, unstable angina, previous MI, previous PCI, previous CVA, left ventricular ejection fraction, multivessel disease, LAD or LMCA as the target vessel, number of treated vessels, number of treated lesions, number of stents, stent length, overlapping stents, reference vessel diameter, basal SS, residual SS, basal ES, residual ES and treatment assignment. That is described in Table 2SA

Stent designs in each arm

BMS group:

Waltz (Microport Inc, Shanghai, China); NexGen (Meril Life, India); Rebel (Boston Scientific, Massachusetts, USA); Pro Kinetic Energy (Biotronic Inc,Berlin, Germany) , Coroflex Blue (Braun Melsungen AG, Germany)

DES group

ORSIRO (Biotronic Inc,Berlin, Germany); Firehawk (Microport Inc,Shanghai, China); Alex-Plus (Balton Inc); SupraFlex Cruz (Sahajanand Medical Technologies, India); Coroflex Isar Neo

(Braun Melsungen AG); YUKON (Transluminal, India); Promus (Boston Scientific, Massachusetts, USA)

Angiographic Risk Scores

Syntax Score (SS) was defined as was initially described and validated by the authors (24). They scored all lesions, including intermediate (≥ 50%) stenosis and lesions located in small vessels(≥1.5mm).

ERACI Score is similar to SS but excluded in the scoring of all intermediate stenosis, and only lesions≥70% were considered and scored.

Any lesions in small vessels (<2.0mm) were not scored.

ISR lesions were not considered in SS, and in ERACI, they were scored as heavy calcification. SS and ES lesion severity and vessel size were visually estimated (24-26).

Usually, fewer lesions are considered high or intermediate risk in the ERACI score compared to the SS, which is applied by baseline or to residual angiographic risk post-PCI.

Causes of Exclusion on the Trial.

2385 patients were excluded. The causes of exclusion were the patient and reference physician's preference for DES use (1473), no candidates for DES or BMS (127), refusal or failure to sign informed consent (511), inability to long-term follow-up (98), contraindications for colchicine (155), and suspicion of COVID-19 infection (21 patients).

14.7% (413/2798) of patients recruited were entered into the study and randomized.
